# University Wellness Program—A Pedagogic Innovation to Nudge Wellness and Sustainability Among Students

**DOI:** 10.3389/fpubh.2022.844024

**Published:** 2022-04-29

**Authors:** Padma Venkatasubramanian

**Affiliations:** School of Public Health, SRM Institute of Science and Technology, Chennai, India

**Keywords:** university, wellness, campus sustainability, pedagogy, future preparedness

## Abstract

Anthropocentric activities have induced climate change, threatened planetary health, and harmed human health and wellness. The changing lifestyles, dietary patterns and digital obsession have affected the mental and physical health, particularly of the youth. University campuses reflect the challenges faced by the society at large and therefore make for an ideal ecosystem to initiate positive changes toward wellness and sustainability. The energy of ~200 million university students globally is largely unleveraged for facing these challenges. Values of empathy and sustainable living are crucial to be inculcated, alongside technical and managerial skills for leading the mass transformation. This article describes a novel pedagogic approach called the University Wellness Program (UWP). The aim of UWP is to equip students with technical and leadership skills to achieve wellness and campus sustainability. That is, UWP is a platform that facilitates the students to design and implement multi-disciplinary projects that address campus related challenges. In the process, they acquire the necessary soft and technical skills to solve real-life problems. The durability of UWP is secured since the projects and activities are explicitly linked to existing curricula and evaluation system of the university. The strategy and framework adopted, and the early experiences of implementing UWP are shared. UWP is amenable for replication globally and has the potential to create change-makers.

## Introduction

Human wellbeing is an outcome of complex, interrelated factors involving the individual, society, and the planet (1). These are captured to a large extent by the UN Sustainable Development Goals (SDGs). Achieving the SDGs requires a holistic approach and presumes values that cut across the goals, but which are not clearly listed. For example, the requirement of an eco-centric mind-set (as opposed to ego-centric), value for the planet, organizational abilities, the vision and leadership qualities etc. are assumed in SDGs. SDGs provide targets and broad guidelines expecting each country to strategize its own plans, monitor and execute them. This would require teams of change-makers including critical thinkers, technical experts, innovators, leaders, and managers for action.

### Anthropocentricity and the Human Wellbeing Challenge

Anthropocentric activities have adversely impacted biotic and abiotic environments, leading to unprecedented changes to climate (2). The latest (Sixth Report) of the Intergovernmental Panel on Climate Change (IPCC) categorically concluded that human activities induced Climate Change and Global Warming, will lead to discernible changes within the next 20 years (3). Frequent outbreaks of zoonotic diseases in recent times including Zika, Ebola and several others are attributed to environmental degradation (49). The ongoing COVID-19 pandemic has reminded us about the interconnectedness of humans with each other, the environment, and other beings on this planet, albeit in an adverse way (4). The COVID-19 pandemic has also negatively impacted the mental health status, exacerbating stress, anxiety, depression, and suicidal ideation, and increased substance abuse (5). There has been a set-back of health and SDG indicators of all countries during the last 1.5 years, due to the COVID- 19 pandemic (6).

Human lifestyle and dietary changes over the last 3–4 decades have seen a steady drop in mental wellbeing and an increase in respiratory and metabolic diseases, cancer, and injuries (7, 8). An analysis of the surveillance data from 105 countries indicates that one fourth of the youth do not follow the public health guidelines for physical activity (9). According to the current figures of the World Health Organization (10), 14% of global burden of diseases is due to mental health disorders and substance abuse, with ~800,000 deaths every year (11). Rising levels of anxiety (42%), depression (36%), suicide ideation (16%) and self-injury (~9%) are growing among university students, in particular (12). A survey on 37,500 university students from 140 universities in the UK found that 1 in 5 students had a mental health issue, and 1 in 3 felt they needed psychological help (13). There could be direct and indirect factors that exacerbate loneliness, stress, depression etc. among young students in university campuses, leading to untoward behavior including substance abuse and suicidal tendencies (14). In the Indian scenario, the stigma associated with seeking professional help for psychological issues, lack of public awareness, and an acute shortage of trained manpower lead to neglect and marginalization of the affected (15). The Mental Healthcare Act (16) of the Government of India was an attempt to mainstream mental health under the National Health Mission in order to ensure better care through the health systems. However, a lot can and needs to be done at the family and educational institutions level to offer the emotional support that the youth require.

Other man-made challenges that threaten the future of mankind largely stem from inequity (49). These include conflicts, war, political marginalization, and migration. Mitigating these challenges require innovative global and local actions in parallel. Leading healthy lifestyles requires learning to “live and let live” and sustainable consumption behaviors. A groundswell of people with awareness, empathy, values, and skills, is required to bring about the transformation. The UN Environment Program has warned of a shortage of skills and manpower for achieving a green and sustainable economy (17). Engaging the youth in influential spaces, not just brings energy and innovation but is crucial, considering they will bear the brunt of the impact of the anthropocentric activities in the future (18).

### Universities-Mini Societies

Around 3.5 billion young adults (<30 years of age) live in this world, which amounts to almost 50% of the total global population (19). Of these ~207 million, a staggering number, are university students (20), whose power is yet to be tapped for addressing serious issues faced by humanity today, concerning climate change, public and planetary health. Ways of constructive engagement with youth are being explored to involve them in decision-making (21). In 2010, the UN General Assembly resolved to involve youth in decision-making and action for a sustainable future (22). The UN recognizes that the youth need to play a significant role in the realization of Sustainable Development Goals (SDGs) as critical thinkers, change makers, innovators, and communicators (23). In 2019, the UN conducted its first Youth Summit in New York to discuss Climate Change and youth participation to achieve SDGs. The SDG Students program, an initiative of the Sustainable Development Solutions Network (SDSN). Youth was launched by the UN SDSN in 2012. This network aims to engage university students globally to achieve the 2030 SDGs agenda as well as to empower them with the knowledge, skills, and pathways to action to be effective change agents (24).

The concept of Health Promoting Universities and a framework for integrating health in curriculum and university system was proposed in 1998 by academicians in the UK (25). The Okanagan Charter proposed a transformative vision for Health Promoting Universities and Colleges in Canada, to strengthen communities and contribute to wellbeing of people and plan (26). The sustainability of these initiatives requires conceptualizing and customizing strategies that integrate contextual research and action programs for wellness and planetary health *within the academic systems* of universities. The University Wellness Program (UWP) is one such pedagogic innovation that evolved independently within the *milieu* of a typical Indian university. This article describes the UWP concept, genesis, the pedagogical framework and the process adopted. It shares the initial experiences and challenges faced.

## University Wellness Program

University Wellness Program (UWP) is an experiential learning platform that encourages students to think, design and implement multi-disciplinary projects for the benefit of the university community. The twin goals of UWP are (i) to achieve wellness and campus sustainability and (ii) to equip students with technical and leadership skills. The vision of UWP is ambitious and its durability is addressed by explicitly linking all its projects/ activities *to existing curricula and evaluation system* of the academic programs in the university. UWP provides the scope for empathy, inclusiveness, reflection, ideation, collaboration, and leadership. Over time it is expected to create an ecosystem of empathy, equity, and value for fellow beings and the planetary resources. The students also become equipped with the necessary practical skills and competencies to tackle real-life problems when they graduate and become future global citizens.

### Genesis of UWP

**Figure 1:**
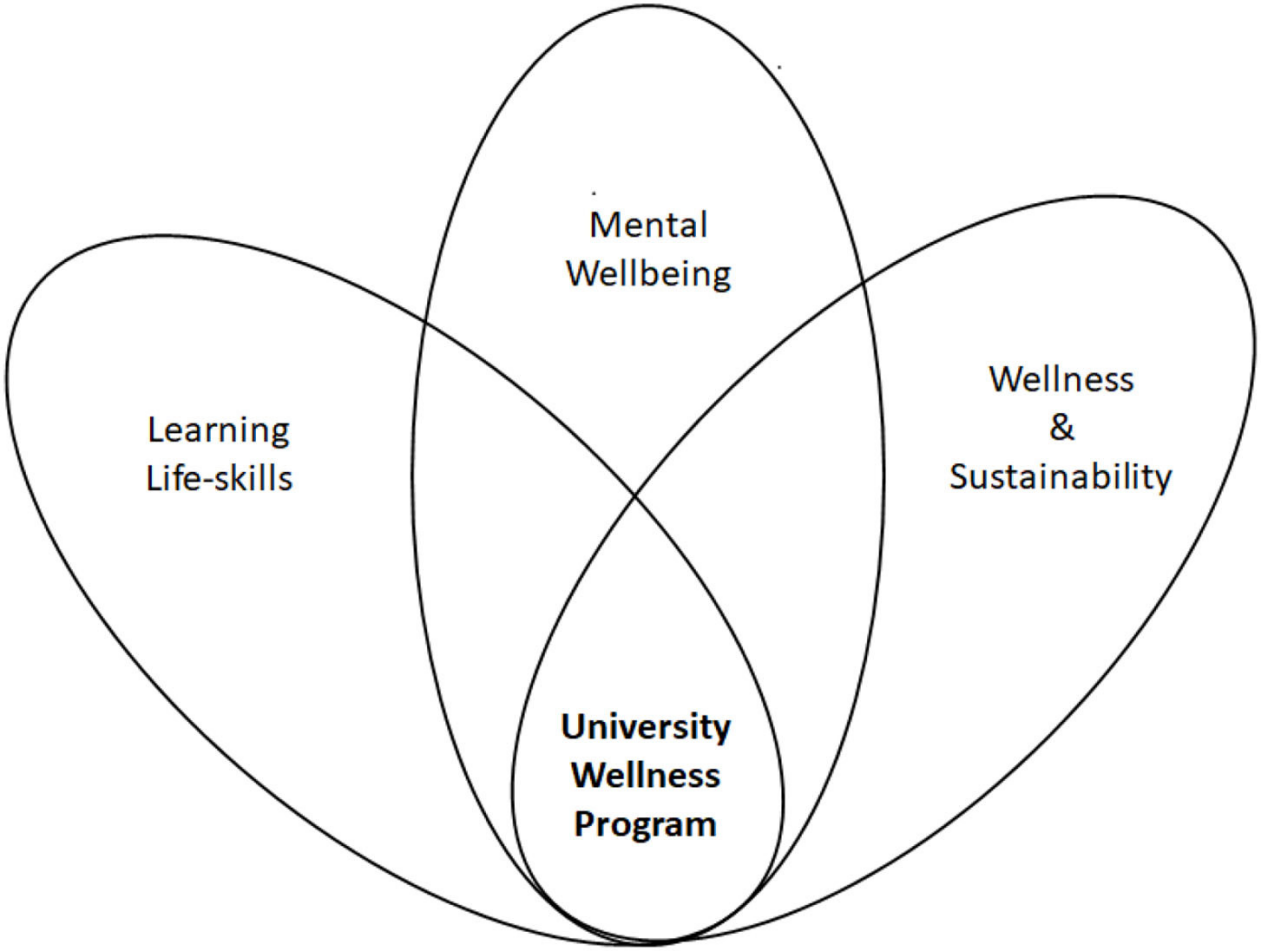
Genesis of UWP.

There were three main thought processes that led to the genesis of UWP during 2019 (Figure 1).

#### Mental Wellbeing

The National Crime Records Bureau (NCRB) reported that young adults in the age group of 18–30 years was the most vulnerable, contributing to >35% of suicides in India. It was very disturbing to note that every hour one university student succumbed to suicide in India (27, 28). Globally, suicide was the second leading cause of death among 15–29-year-old, next to road accidents (10). The American College Health Association reported that 26% of the students surveyed were feeling so depressed that they could not carry out their normal functions (29). The world over, the Happiness Index had decreased in >50% of the countries surveyed (30), with mental health becoming a significant concern. These scenarios, wherein young adults and students were succumbing to poor mental wellness, indicate a deep-rooted malaise in our societies. The fundamental reasons for this need to be explored and addressed. Students are critical to the progress of any nation and the future of mankind, and their wellbeing is the collective responsibility of all concerned.

University freshers struggle to adjust to academic challenges, new environments, and relationships. Facing these challenges requires a positive mental framework and resilience. Moeller et al., point out that emotional and social quotients (EQ and SQ) play an equal role as the intelligent quotient (IQ) in a student's academic performance (31). EQ and SQ help them in understanding, expressing and managing emotions appropriately (31).

#### Wellness and Sustainability

Tens of thousands of people move around in a typical university campus every day. For example, there is a footfall of ~100,000 people including faculty, staff, patients in university hospitals, vendors and ~50,000 students mill through a typical multi-disciplinary university campus in India (32). Therefore, universities are strategic places to begin the wellness drive and to “walk the talk” about wellness and sustainability. Several universities are already actively engaged with achieving SDGs through management action, organizing events, student projects etc. Green metrics and recognition of universities through awards have been instituted to rank the universities globally (33). These initiatives do bring about awareness and positive changes on campus (34), but since they are top-down approaches and external to the campus, they tend to be addressed in a non-strategic and *ad hoc* manner and become yet another indicator in the university ranking system. For any effort to become embedded in the system and to change the mindset and behavior of the stakeholders, requires a parallel, contextual, bottom-up approach as well, involving the key stakeholders, along with programmatic interventions that are sustainable by design (35).

#### Learning Life-Skills

A significant trigger for the genesis of UWP was the question as to whether the graduates are future-ready (not just job-ready). The graduating students need to also be prepared to face the global challenges of climate change, conflicts, and disasters. While being sensitive and empathetic to societal issues, the graduates need to develop the technical and leadership skills to address these challenges. It was reported that almost every other university graduate from Indian universities in 2018 did not have the necessary skills (36). Practical, evidence-based solutions that are appropriate to the community/environmental contexts need to be developed. Acquiring soft skills of communication, collaboration, and negotiation, will prepare them to navigate systems. This requires the teachers and the university management to be clued-in to contemporary global and local issues like climate change and conflicts. It also requires a pedagogy that has adaptable teaching and evaluation system that not just assesses the technical skills but also the students' critical thinking and ability to apply theoretical knowledge for practical solutions for contemporary issues.

Higher education is not just for better jobs and pay packages for the graduates but also to create thought leaders and change makers for human development and a sustainable future (37). A study by Entwistle and Peterson reports that students in higher education who seek deeper meaning tend to perform better than those who see it as instruments for better jobs (38). However, majority of the students are not trained in critical thinking or skilled for solving real-life, complex problems (39).

Thus, with enough complex issues on campus, a university would be an ideal place to implement UWP.

### UWP- Why a “Wellness” and Not “Health” Program?

The commonly accepted definition of Health is that of WHO. Health is a “state of complete physical, mental and social wellbeing and not merely the absence of disease and infirmity” (10). In the context of UWP, “wellness” refers to *individual's perception* of being well. The feeling of “being well” is important for carrying out routine functions and in living life to the fullest. One can clinically be perfectly “healthy” but still not feel “well”, while another who has serious health problems can still “feel” well, enthusiastic, and active. Wellness is dynamic and dependent on individual mind-set and the ability to bounce back from any obstacle.

The Global Wellness Institute defines wellness as “the active pursuit of activities, choices and lifestyles that lead to a state of holistic health”. That is, wellness can be aimed for and actively pursued (40). Wellness is captured succinctly in Indian health traditions by the Sanskrit term *Svasthya* (*sva-*individual*, sthya-*stability). That is, the state of being in harmony with one-self. This state would vary with person and context. It was felt that “wellness” may be a better terminology to be used in a university context than “health” or “healthy”. Therefore, the **University Wellness Program** or **UWP** was coined.

## Pedagogical Format

**Figure 2:**
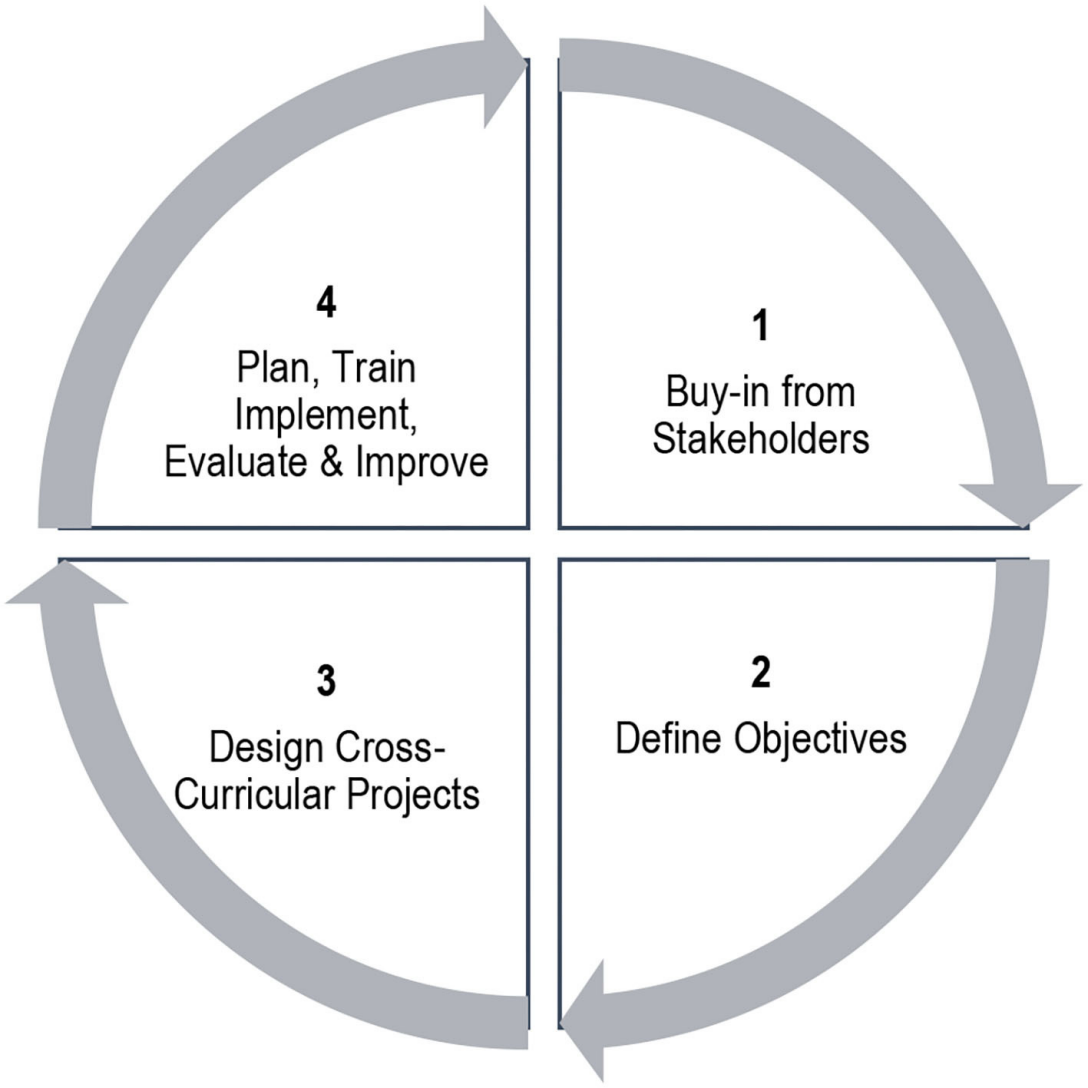
Framework to initiate University Wellness Program.

UWP being a new concept to Indian universities, the challenge was to create processes that aims to achieve wellness and campus sustainability through student learning and doing. The challenge was also to integrate UWP within the university system for better programmatic sustainability. UWP kick-started with an evolving strategy, following four broad steps as given in Figure 2.

Almost all university academic programs have structured syllabi comprising of both theory and practicals/ practicum which usually have a one-to-one correlation, with the practicals strengthening theoretical learning. For example, a Chemistry student gets exposed to the theory of acids and bases during lectures, and learns better by performing acid-base titrations during practicals. These are absolutely essential and should continue. However, they are typically stand-alone, mono-disciplinary learning methods that most often are not linked to real-life, multi-disciplinary issues. The mainstream model of education at best produces students who learn, recall and predominantly practice mono-disciplines post their graduation. Schön (50) describes the much needed “reflection-in-action” method, to provide scope for student learning and practicing on real-life issues, while on campus.

One of the ways a broader application of theoretical learning can happen is by making UWP an integral part of existing curricula of the various academic programs. The methodology is iterative involving discussions with key stakeholders. The framework adopted to initiate UWP and early experiences have been shared in the following sections.

### Buy-In From Stakeholders

**Figure 3:**
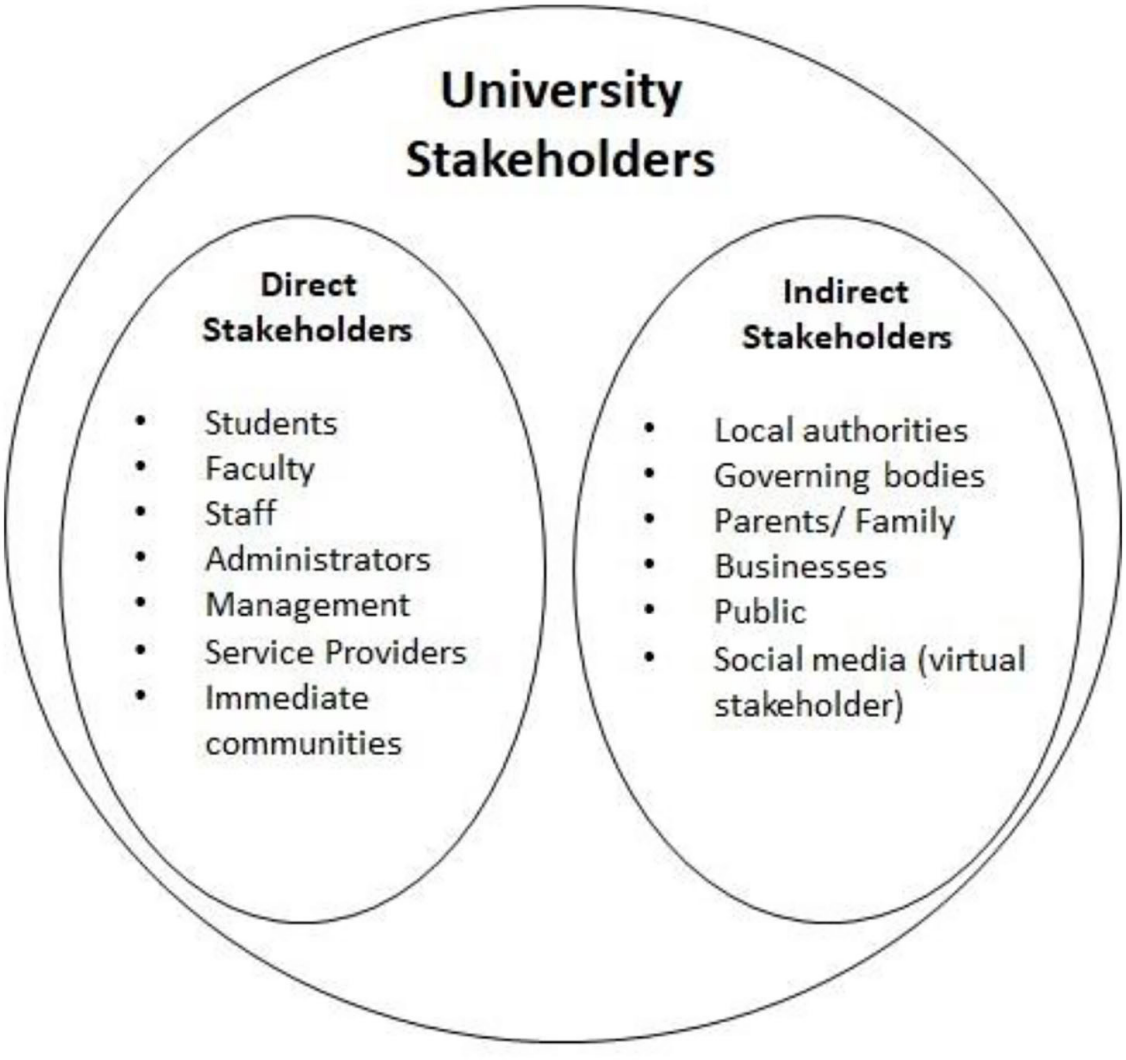
Typical stakeholders in a university/HEI.

Freeman's simple definition of “stakeholder” as “any group or individual that is affected by or can affect the achievement of an organization” is applicable in a university setting (51). University stakeholders can be of two kinds, direct or indirect stakeholders (Figure 3). Direct stakeholders in a university campus include students, teaching, non-teaching and administrative staff, the management, housekeeping, other service providers, and the immediate communities outside the university campus etc. whereas indirect stakeholders include those who may not be visible on a daily basis or be present on campus but yet influence campus wellness and sustainability. The latter include the institutional, local and other governing bodies, parents/family of students, other universities, businesses and the public at large. In today's times, the social media and network play important roles as virtual “stakeholder” group, especially in a student's life (41, 42).

Without the buy-in from key stakeholders, any new program can run into multiple roadblocks during implementation. Therefore, one of the essential steps to be taken before initiating any program is to obtain the buy-in from key stakeholders, particularly from the top management during the initial stages (43).

In the case of UWP, sharing the vision, objectives, and scope helped gauge the readiness of the different stakeholder groups to adopt the program and to enthuse their participation. It also helped garner the necessary administrative, financial, and human resource support from the management to initiate the program. Meetings with the faculty and heads of departments provided an opportunity to brainstorm ideas, learn about ongoing initiatives on sustainability and wellness, and the major implementation challenges faced. Interactions with student groups were useful in providing insights into the kind and intensity of issues on campus and assessing the capacities and interest of students. The faculty and students at the School of Public Health (SPH) were the immediate stakeholders, who in turn carried the UWP agenda forward.

### Define Objectives

Defining the objectives and setting targets is crucial for the success of any project because it brings clarity and transparency to all. Drucker in his 1954 book, “Practice of Management” coined the term *Management by Objectives* that describes the importance of all stakeholders to participate in defining the objectives for a program's viability and durability (44). Goal-setting guides projects and provides the scope for monitoring performance and measuring success. It also is a motivational lever for the team (45).

The Postgraduate (PG) and Undergraduate (UG) academic programs at the University run on a semester basis and therefore it was decided to set semester-wise (5 months) objectives. Brainstorming meetings with the management, faculty and students were held before crystallizing the objectives for UWP for the first semester. Two mini projects were undertaken by the students and faculty of SPH, for the first semester (August–December 2019):

Mini Project #1: Launch of UWP

In order to announce UWP, it was decided to hold a formal event inviting high profile individuals, the top management, heads of departments, faculty and students on campus. It was also decided to invite the media and press for giving the event good visibility.

Mini Project #2: Identification of key student issues

Students were the largest (~60–70%) stakeholders on university campus. Hence it was decided to identify issues of this group, to begin with. It was important to know the status of wellness of the students, the key issues faced by them and the coping methods.

The framework and processes were crystallized and documented for initiating the two mini- projects during this period.

### Design Cross-Curricular Projects Linked to Curriculum

Practice-based learning is an essential part of the pedagogy of professional academic programs. For example, a student training to be a doctor, dentist or a nurse is exposed to patients on a regular basis, so that they learn how to treat patients when they become full-fledged practitioners. This is not the norm in other disciplines and the students are rarely exposed to wellness or sustainability concepts and actions.

The UWP idea is to encourage students to look inward, within the campus and their immediate environments including the communities, to identify and address the issues, leveraging their respective domain skills. For example, a Visual Communication student from the Humanities and Sciences College, who learns the skills in photo/video-graphy, drawing, computer graphics, recording etc. and a public health student from the Health Sciences College, who learns health promotion, communication, nutrition, and research methods etc. as a part of their respective academic syllabi, can work together to identify the nutritional status of students on campus and to create effective health promotional materials. This collaboration would bring about a better researched audiovisual and there would be a cross-fertilization of ideas. Thus, the practical application of theoretical learning will be for achieving overall campus wellness.

Once the aim is stated, designing the UWP projects three main steps were involved (Table 1):

(i) *Detail the activities*: Once the objective(s) of the project were set, the broad activities that would be required for fulfilling the objectives were delineated. For example, we wanted to create and inaugurate a logo for UWP during the UWP launch event, which meant designing the logo was one of the UWP activities under Mini-project #1.
(ii) *State the Learning Outcomes:* Identify the technical and other skills that are required to achieve the project learning outcomes. For example, knowledge about effective communication and branding, and skills to design a logo are some of the expected learning outcomes from Mini Project #1.
(iii) *Map and Select Modules*: Select those modules from the syllabus whose learning objectives match with those of the UWP project outcomes. Leverage modules that provide the space to learn hands-on technical skills and/or soft skills required to achieve the targets (Table 1). The module should also have the scope for assessing the students' performance as a part of the regular evaluation system.

**Table 1:** Mapping of modules and skills and competencies to achieve the targets.

**Define the objectives**	**Detail the activities**	**State the learning outcomes from the project (technical and soft skills)**	**Map the modules** **(pertinent to the listed skills/competencies)**	
Official launch of UWP (Mini-Project #1)	•Design UWP logo •Plan, monitor and execute the launch event •Archive the project outputs	**Technical** •Designing UWP logo •Planning, execution, monitoring and management of event **Soft skills** •Leadership, teamship •Interpersonal and communication skills	**Module name** •Social and behavior change, effective communication in healthcare •Health management: management principles and practices •Communication skills	**Expected learning outcomes of modules** •Application of concepts of strategic communication in Public Health •Planning and management •Public health leadership •Public speaking, writing, inter-personal skills
Identification of key student issues (Mini Project #2)	Conduct research to identify •student wellness (physical, mental) •key issues faced on campus •coping methods and •recommendations	**Technical** •Research skills and aptitude •Planning, monitoring; execution and management of project **Soft skills** •Leadership, teamship, interpersonal and communication skills	•Principles of research methods •Communication skills	•Knowledge and practice of types of research methods •Protocol design and ethical process •Data collection, analysis and interpretation •Research communication skills •Public speaking, scientific presentation, inter-personal skills

Broadly, the skills and competencies required for implementing the Mini-projects were *explicitly spelt out* as “learning” outcomes and tagged to pertinent module(s), and the students were formally evaluated for the same. Sometimes when the targets set required multi-disciplinary efforts, it was important that departments within or outside the college collaborate to achieve them.

### Plan, Train, Implement, Monitor, Evaluate, and Improve

#### Plan

While Mini Project #1, namely the UWP launch, was an event that required branding, networking and managerial skills, Mini Project #2 (Identification of key issues) required mainly research skills. In consultation with the SPH team of faculty and students, plans were developed.

**Figure 4:**
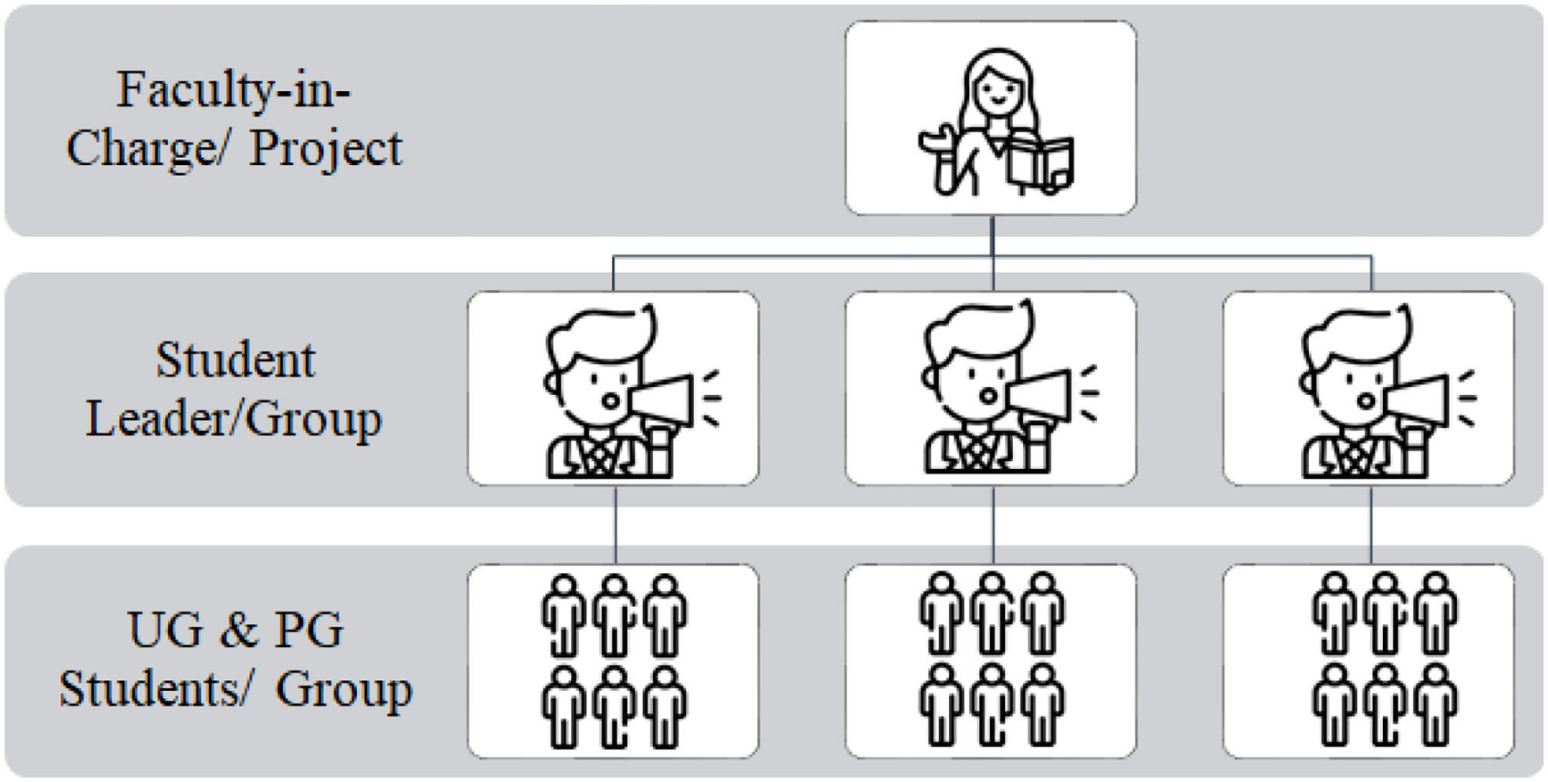
Grouping of students for UWP projects.

The groups, the milestones, end points, timelines and responsibilities to implement the two mini projects, were written down and shared with all. The PG and UG students across all years and programs, were vertically grouped. That is, a I year UG student who was freshly out of high school got to observe and learn from the final year senior PG students, and the seniors in turn learnt empathy and tolerance. The students selected one leader/representative from each group and one faculty volunteered to drive each of the UWP projects (Figure 4). The main responsibility of the faculty-in-charge and the student leaders was to drive and monitor progress. Wednesdays were set aside as UWP days when all the students (~130 nos.) and faculty came together to review the progress of the two mini-projects and change strategies as required.

#### Train

Students were trained and guided on the technical skills and competencies required to execute the activities. The faculty handling the module(s) has the liberty of selecting the aspect(s) for which training is required and this would depend on the set objectives and activities. It may or may not be the regular training provided. For example, as a part of the ‘Social and Behavioral Change and Effective Communication’ module, a professional creative designer was especially brought in to conduct a workshop on the importance of brand building. The students learnt the essential features of branding for public health. Creating a logo for UWP was given as an assignment for which they were evaluated and the team which designed the best logo for UWP was to be rewarded by show-casing it at the UWP launch event. It was declared that in recognition of the students' participation, certificates for leadership and contribution would be distributed.

The theoretical aspects of the principles of Research Methods (types—qualitative, quantitative, and mixed-methods), protocol design, ethics, data collection, analysis and interpretation etc are taught as a part of the regular teaching of the module. However, practical training was also provided on qualitative research including performing Free Listing, Focus Group Discussions (FGDs) and analysis of qualitative responses, which were essential skills to fulfill Mini-Project #2.

Students were oriented in soft skills including listening, team work, tolerance and public speaking as a part of the “Social and behavior change, effective communication in healthcare” module. They were nudged to reflect on the process and recommend next steps.

Thus, over and above the regular teaching, special training was given depending on the skills and competencies required for fulfilling the project. It may vary depending on the project requirement.

### Monitoring and Evaluation

For durability of UWP it is important to leverage existing university evaluation system and integrate the student projects within its scope. For example, in our university system 40% of marks is toward continuous internal assessment and 60% allocated for end-semester examination. The internal assessment system provided adequate scope for evaluating the practical learning of the students in the UWP projects. Normally, assignments, presentations and cycle tests on theoretical learning are evaluated in internal assessment. For UWP, execution of project activities and deliverables became part of the existing continuous, monitoring and internal assessment system of the module(s).

Students were evaluated for individual as well as group contributions for the fulfillment of two projects. Soft skills such as leadership qualities, communication skills, empathy, inclusivity and teamship were encouraged and assessed based on class interactions and presentations during the semester. The projects themselves were evaluated by the success of fulfilling the objectives, namely UWP launch event and prioirtization of key issues faced by the students. We are in the process of creating the framework for evaluating UWP at the university level. Five year goals, roadmap and key success indicators are being developed.

### UWP Project/Activity Criteria

Research and action projects, events and activities are a regular part of any univerisity. However, keeping in mind the UWP objectives and durability, a set of criteria were developed by us. Only those projects/activities that fulfilled these criteria were qualified as a UWP project/activity.

(i) **the purpose/objective** has to be clearly spelt out and should contribute to wellness and/or campus sustainability
(ii) **the expected outputs**/**outcomes stated**- these could include products/service/events etc. Other practical/soft skills learnt need to also be indicated
(iii) **the student academic program/modules and evaluation methods** that will be leveraged need to be mentioned
(iv) **the faculty in-charge** who will drive the project/activity, needs to be named
(v) **name the student leader and team(s)** who will execute the project, need to be named
(vi) **the skeletal plan** of execution needs to be secured

A project was considered successful if the set objectives and learning outcomes were achieved, fulfilling the above criteria.

## Initial Experience

### Mini Project #1: UWP Launch

**Figure 5:**
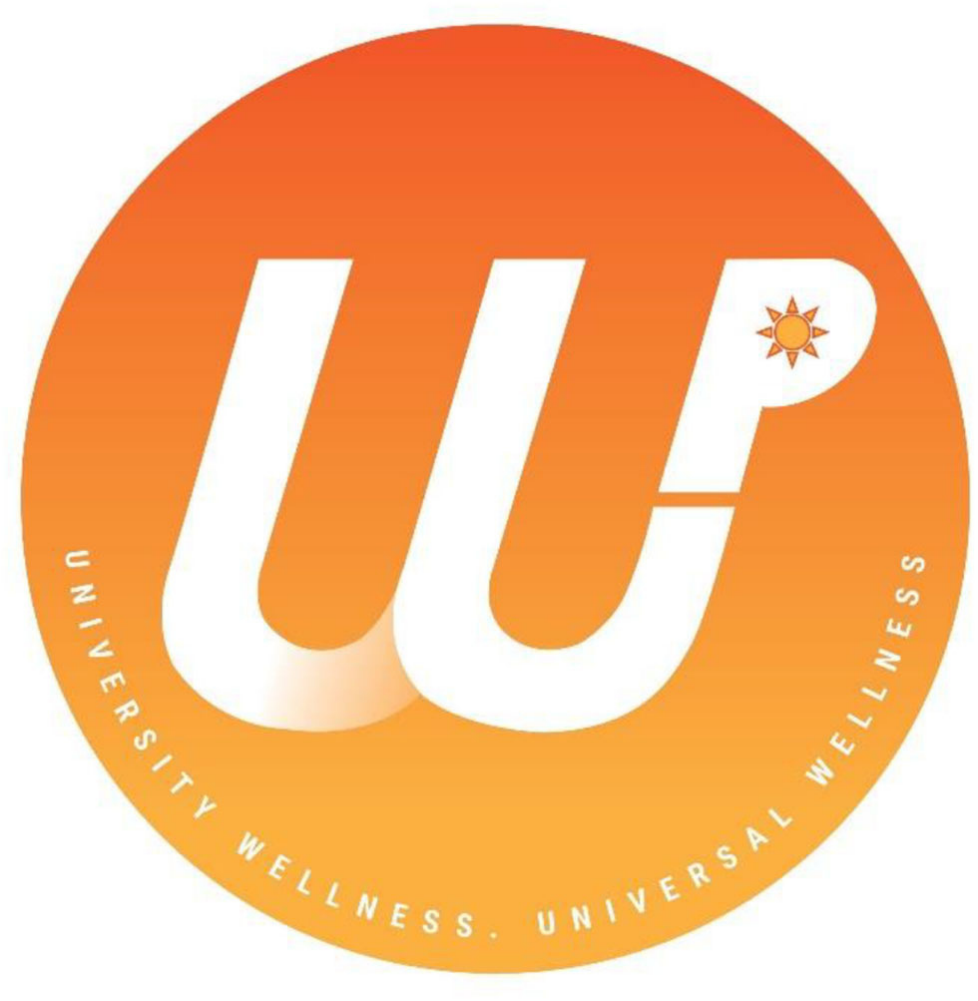
UWP logo. MPH students designed the logo and learnt the importance of branding during the “social and behavior change, effective communication in healthcare”.

UWP was successfully launched at an official event organized on November 12th, 2019, and the best logo for UWP that was designed by students was unveiled at the event (Figure 5). The group which designed the best logo got an opportunity to describe the logo and the tagline at the event. The bright orange shade in the logo represents positive energy, youth and action. The tagline “University Wellness. Universal Wellness” indicates the vision that a small change in university can lead to changes at the global level and the reach of the universities. The UWP launch event was planned and managed by the students. Dr. Kasturirangan, Chairman of New Education Policy Committee (2019), Government of India presided over the function. It received good coverage in social media and press. The aim of launching UWP, designing the logo and to do it through students as a part of existing academic evaluation system, was achieved.

### Mini Project #2: Identification of Student Issues

The students received hands-on experience in conducting qualitative research under the Research Methodology module (Table 1). They learnt to perform free-listing, FGDs, coding and categorization of qualitative responses. Four key themes emerged from the student responses with important sub-themes (Table 2).

➢ Mental Wellbeing—stress, anxiety, depression and suicidal ideation thoughts etc emerged as important emotions that affected the students daily performance. Girl students specifically mentioned pre-menstrual syndrome (PMS) as an issue
➢ Lifestyle behavior—social media time, mobile usage, substance abuse, food intake, sedantary lifestyle, indisciplined sleep pattern etc. were ackmolwledged as significant aspects of student life on campus
➢ Water, Sanitation and Hygiene (WASH)—campus WASH facilities were mentioned as being important. Frequent cleaning of toilets was expressed as important during busy times. The girl students mentioned that better and accessible facilities can be provided, to help during menstruation
➢ Global Warming—It was important to note that global warming, in general, came up in the FGDs as an important issue that the students were concerned about. These included pollution, food wastage, transport, use of plastics and paper, within and outside the campus. The FGDs strongly highlighted that the students realized the negative impact of human (anthropocentric) activities on the health of communities and the planet. There was a will to contribute and make a difference

**Table 2:** Themes and sub-themes of key student issues that emerged through free-listing and FGDs.

**Themes**	**Sub-themes**
Mental health	Stress, anxiety, depression and suicidal thoughts, Pre-Menstrual Syndrome among girls
Lifestyle behavior	Social media time, mobile usage, substance abuse, food intake, sedantary lifestyle, sleep pattern etc
WASH	Frequency of cleaning of toilets, better facilities for girl students
Global warming	Pollution, food wastage, transport, use of plastics and paper, within and outside the campus

The presentation of the research findings of the key issues faced by the university students at the UWP launch (i.e., Mini-project #1) was a highlight of the event and was much appreciated by the management and audience.

Going forward, prioritization of projects and activities to be taken up needs to be worked out. This would depend on the urgency, resources available, and the interest of the students, faculty and management.

## Discussion

The Okanagan Charter started the Canadian Health Promoting Campuses Network in 2015 that called for embedding health in all aspects of campus culture (26). The UK Healthy Universities Network promoted a similar concept (46). The UWP idea emerged independently in 2019, within the academic, socio-cultural and political context of a typical Indian private university.

This article has introduced a new pedagogic concept called the University Wellness Program (UWP), which enables students to work toward achieving wellness and campus sustainability. Through the implementation of the two Mini projects, a methodology/framework has been delineated to demonstrate how UWP can be embedded in university learning and evaluation system. One project was to formally launch UWP and the other was to identify and prioritize key issues faced by students on campus.

The UWP Mini-Projects provided the students hands-on technical skills, managerial, leadership and other soft skills to implement the projects. Over and above the technical skills required in implementing the projects, the students learnt listening skills, empathy, ability to think critically, leadership and team-ship, public speaking and effective communication. These qualitative aspects were monitored and assessed on a weekly basis by faculty. All teams and presented their work at the final project presentation, as college seminar. The best teams got opportunity to present at the UWP launch event.

We expect that the impact created by UWP *per se* will become evident only over a period of time, say 5 years. A system for longitudinal research is being set up with the current cohort of students. A database is planned to be created for capturing every student's wellness at the university level. We have also established a core UWP committee consisting of experts and student representatives from different departments across the university to look at the various UWP aspects, including research/action/fund mobilization/management/communication related. The committee members are the drivers to keep UWP going. A 5 year roadmap for UWP is being created, with goals and key success indicators. Faculty and students of SPH and the Department of Computer Science & Engineering are creating a digital platform and a dashboard to capture the UWP projects and progress. This in itself was taken up as a UWP project. These are some of the work in progress beyond 2019.

**Figure 6:**
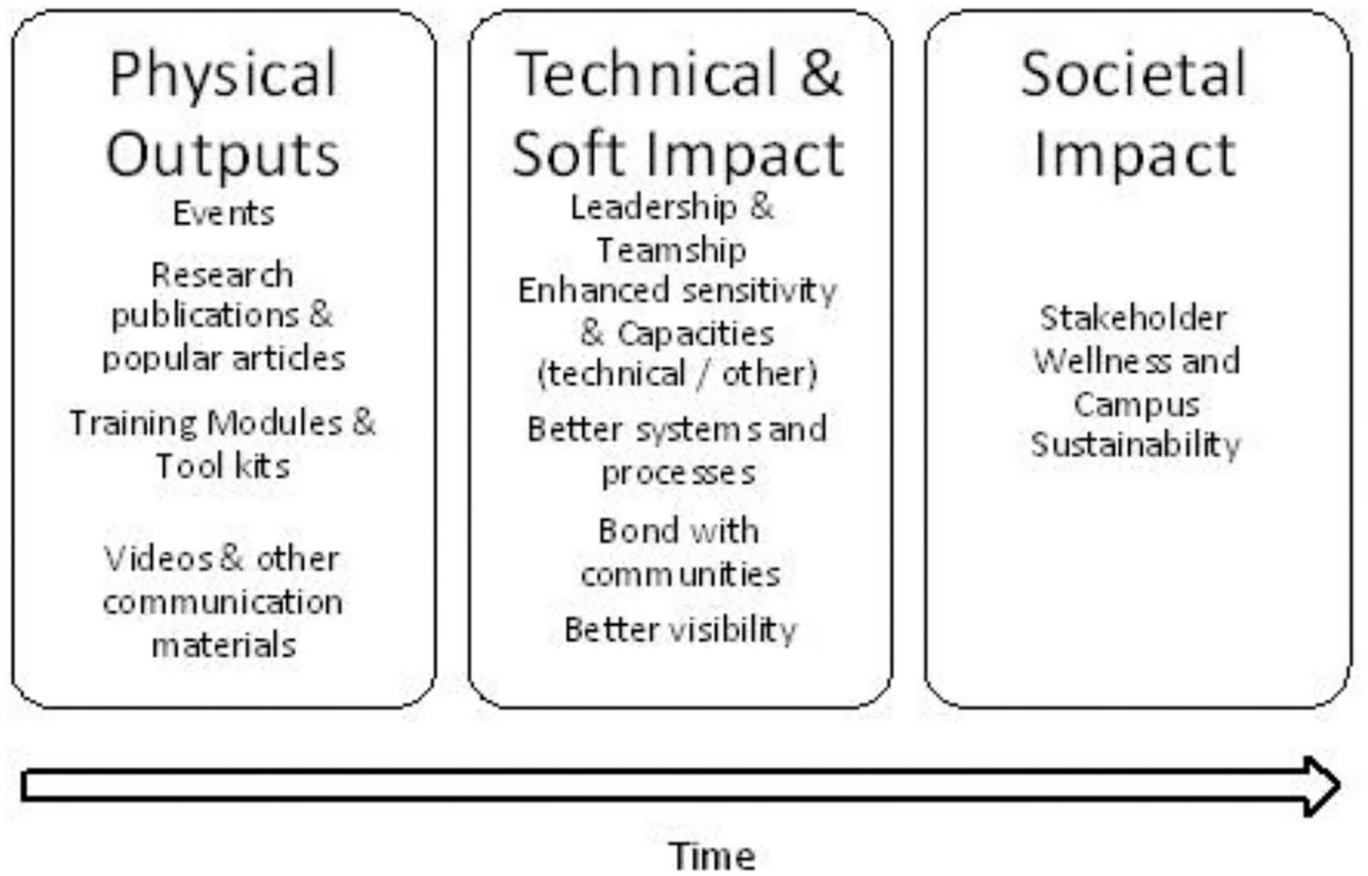
Expected outputs from UWP over time.

The expected outcomes from UWP over time can be classified as physical outputs, technical and soft impact, and societal impact (Figure 6). UWP would provide opportunities for students to have a more rounded development.

### Challenges Faced

The seven factors identified by Valaitis et al., that influence the success of new or existing programs are very relevant for the durability of UWP (47). The factors include (i) clear mandates, vision and goals (ii) strategic coordination and communication mechanisms (iii) formal organizational leaders as collaborative champions (vi) collaborative organizational culture (v) optimal use of resources (vi) optimal use of human resources and (vii) collaborative approaches to programs and services delivery.

The challenges faced in UWP implementation were mainly faculty and program implementation related. Initially, convincing the faculty about the idea took some time. The main reasons for this were (i) resistance to new ideas; as is usual in any pre-existing group (ii) lack of clear-cut methodology to integrate UWP in existing teaching and evaluation system in the early stages of UWP (iii) faculty perception as additional burden to already hectic academic and research schedules (vi) fear of failure and (v) indifference and hesitancy in ownership.

UWP uses practice/problem-based learning (PBL) approach for achieving wellness and sustainability on university campus. PBL has the advantage of imparting capacities among students at the conceptual, practical and attitudinal levels but it presents challenges to the teachers. Loss of control over their teaching styles, selection of topics of students interest, unpredictability of outcomes, fear of exposure of their knowledge gaps and need for more time investment were some of the perceptions of teachers about PBL (48). These issues/fears will need to be addressed to obtain faculty participation, along with training them in the new pedagogy.

Recruitment of a new Teaching Associate who is dedicated for UWP coordination helped immensely in reducing the faculty burden. The faculty buy-in was achieved through frequent meetings and interactions, setting objectives, drawing up clear plans of execution and engagement. Discussing the potential advantages, and outcomes motivated them a great deal.

Identifying topics that were pertinent to key stakeholder issues, campus wellness and sustainability and designing research or action-oriented projects that leveraged existing academic programs required asking fundamental questions. For example, what are the key issues faced by students and the status of wellness of students? What needs to be done and how to crystallize them requires individual and group brainstorming. These were difficult to achieve in an already packed teaching-learning-evaluation university system. We solved these to a large extent by setting aside 1 day in a week for UWP related activities. Explicit linkage of objectives to ongoing modules and the freedom to assign practical work, can enhance the quality of teaching and learning. Also, a campus wellness/sustainability related issue gets addressed in the process.

UWP members become potential stewards and change-makers in university campuses. The activities can also become part of regular clubs, roping-in students and faculty from departments and universities. New academic programs and PhDs could stem from the UWP projects. Toolkits and online/offline training programs can be developed for sharing with other universities. UWP is work in progress and the strategy would need to be modified depending on context.

## Conclusion

The article introduces University Wellness Program (UWP), a novel pedagogical concept to focus on wellness and sustainability issues that face us today. It provides the scope for practice-based learning for students, by leveraging existing academic modules and evaluation system. Our strategy demonstrates the feasibility of UWP. Faculty interest and leadership are probably the biggest assumptions for UWP's durability.

## Data Availability Statement

The original contributions presented in the study are included in the article/supplementary material, further inquiries can be directed to the corresponding author/s.

## Author Contributions

The author confirms being the sole contributor of this work and has approved it for publication.

## Conflict of Interest

The author declares that the research was conducted in the absence of any commercial or financial relationships that could be construed as a potential conflict of interest.

## Publisher's Note

All claims expressed in this article are solely those of the authors and do not necessarily represent those of their affiliated organizations, or those of the publisher, the editors and the reviewers. Any product that may be evaluated in this article, or claim that may be made by its manufacturer, is not guaranteed or endorsed by the publisher.
